# Novel radiohybrid PET-Tracers for SST_2_-targeted imaging of neuroendocrine tumors

**DOI:** 10.1007/s00259-025-07638-9

**Published:** 2025-10-23

**Authors:** Lennard Wendlinger, Mara Parzinger, Alexandra Litvinenko, David Viertl, Philipp Spatz, Alexander Wurzer, Hans-Jürgen Wester, Margret Schottelius

**Affiliations:** 1 Translational Radiopharmaceutical Sciences, Centre Hospitalier Universitaire Vaudois, Lausanne, 1005 Switzerland; 2 AGORA, Pôle de recherche sur le cancer, Lausanne, 1005 Switzerland; 3 Pharmaceutical Radiochemistry, Technical University of Munich, Garching, 85748 Germany; 4 Scintomics Molecular, Applied Theranostics Technologies GmbH, 82256 Fürstenfeldbruck, Germany

**Keywords:** PET imaging, Radiohybrid, DOTA-TATE, Fluorine-18, Gallium-68

## Abstract

**Purpose:**

^68^Ga- and ^177^Lu-labeled theranostic companion tracers have become a mainstay in the clinical management of SST_2_ overexpressing neuroendocrine tumors. Despite the excellent radionuclide characteristics of [^18^F]fluorine for PET imaging, ^18^F-labeled SST_2_-targeted tracers remain underrepresented. Novel radiohybrid SST_2_-tracers with DOTA as a bridging unit were designed, allowing radiolabeling with either ^18^F or ^68^Ga.

**Methods:**

Seven DOTA-TATE derivatives (rhTATE1/2: *N*-SiFA*lin*-*N*,* N*-Me_2_-Gly-d-Dap/Lys(*trans*-DOTA-TATE)-OH and (rhTATE2.1-2.5: H-AA1-AA2-AA3-d-Dap(*N*-SiFA*lin*-*N*,* N*-Me_2_-Gly)-d-Lys(*trans*-DOTA-TATE)-OH) with different linkers and hydrophilic modifiers (AA1-AA3) were synthesized and compared to [^18^F]SiTATE. Competitive binding studies (IC_50_) were performed using hSST_2_-CHO cells and [^125^I]TOC. Internalization was investigated using AR42J cells. Biodistribution and PET/CT studies were performed using AR42J xenograft bearing CD1 nu/nu mice. SST_2_ specificity was confirmed in a blocking study (+/- co-injection of octreotide).

**Results:**

While the first-generation compounds showed good affinity (IC_50_: [^nat^Ga]rhTATE1: 5.6 ± 1.4 nm, [^nat^Ga]rhTATE2: 5.7 ± 0.2 nm) but high lipophilicity (LogD_pH=7.4_ = -1.03 and − 1.19), the inclusion of hydrophilic modifiers ([^nat^Ga]rhTATE2.1-[^nat^Ga]rhTATE2.5) improved affinity (IC_50_: 2.6 to 3.7 nm) and hydrophilicity (LogD_pH=7.4_ = -2.30 to -2.12). The compounds demonstrated efficient internalization (357% to 841% compared to [^125^I]TOC), and variable human serum albumin affinity (84.8% to 98.8%). [^18^F][^nat^Ga]rhTATE2.2 showed the highest tumor accumulation (27.9 ± 4.8%iD/g), while [^18^F][^nat^Ga]rhTATE2.5 showed lower tumor uptake (18.6 ± 6.2%iD/g), but substantially lower background accumulation, providing improved tumor-to-organ ratios.

**Conclusion:**

This study demonstrates a SST_2_-targeted radiohybrid concept using bifunctionalized DOTA as a bridging unit. *N*-terminal modifications with hydrophilic tripeptides improved the pharmacokinetic properties. [^18^F][^nat^Ga]rhTATE2.5 (d-Glu-d-Glu-d-Glu) compares particularly well to [^18^F]SiTATE regarding background clearance and tumor accumulation, with the additional advantage of radiohybrid radiolabeling.

**Supplementary Information:**

The online version contains supplementary material available at 10.1007/s00259-025-07638-9.

## Background

Neuroendocrine tumors (NETs) are a heterogeneous group of neoplasms originating from the neuroendocrine system [1], representing only 0.49% of cancers [2]. NETs often overexpress the somatostatin transmembrane receptor 2 (SST_2_), constituting the basis of SST_2_-targeted clinical theranostics using Tyr^3^-octreotate (TATE)-based companion tracers such as [^68^Ga]DOTA-TATE (NETSpot^®^) and [^177^Lu]DOTA-TATE (Lutathera^®^) [3].

Clinical diagnostics primarily rely on ^68^Ga-labeled peptides, despite ^18^F having superior positron emission tomography (PET) properties - longer half-life (109.7 min), higher β^+^ branching ratio (96.7%) and lower β^+^ energy (0.63 MeV), with the latter providing improved image resolution compared to ^68^Ga (β^+^ energy: 1.90 MeV). The continued use of ^68^Ga tracers stems mainly from historically simpler labeling procedures, whereas ^18^F-labeling was limited by complex, multistep protocols such as [^18^F]-fluoroacylation ((2-[^18^F]fluoropropionyl-(d)phe^1^)-octreotide [4]; Gluc-Lys([^18^F]FP)-TOCA [5] or oxime ligation (Cel-*S*-Dpr([^18^F]FBOA)TOCA [6]). More innovative one-step procedures involve Al^18^F^2+^-complexation by 1,4,7-triazacyclononane-1,4,7-triacetic acid (NOTA; [^18^F]AlF-NOTA-octreotide (OC) [7]), [^18^F]AlF-NOTA-JR11 [8]) or isotopic exchange reactions using ammoniomethyl trifluoroborate (AMBF_3_) of silicon fluoride acceptor (SiFA)-based precursors ([^18^F]AMBF_3_-TATE [9], [^18^F]SiTATE = SiFA*lin*-Asn(Ac-NHβGlc)Asp_2_-PEG_1_-TATE [10, 11]). [^18^F]AlF-NOTA-OC [12], and [^18^F]SiTATE [13] have advanced into clinical application, demonstrating comparable or superior imaging sensitivity to [^68^Ga]DOTA-TATE.

**Fig. 1:**
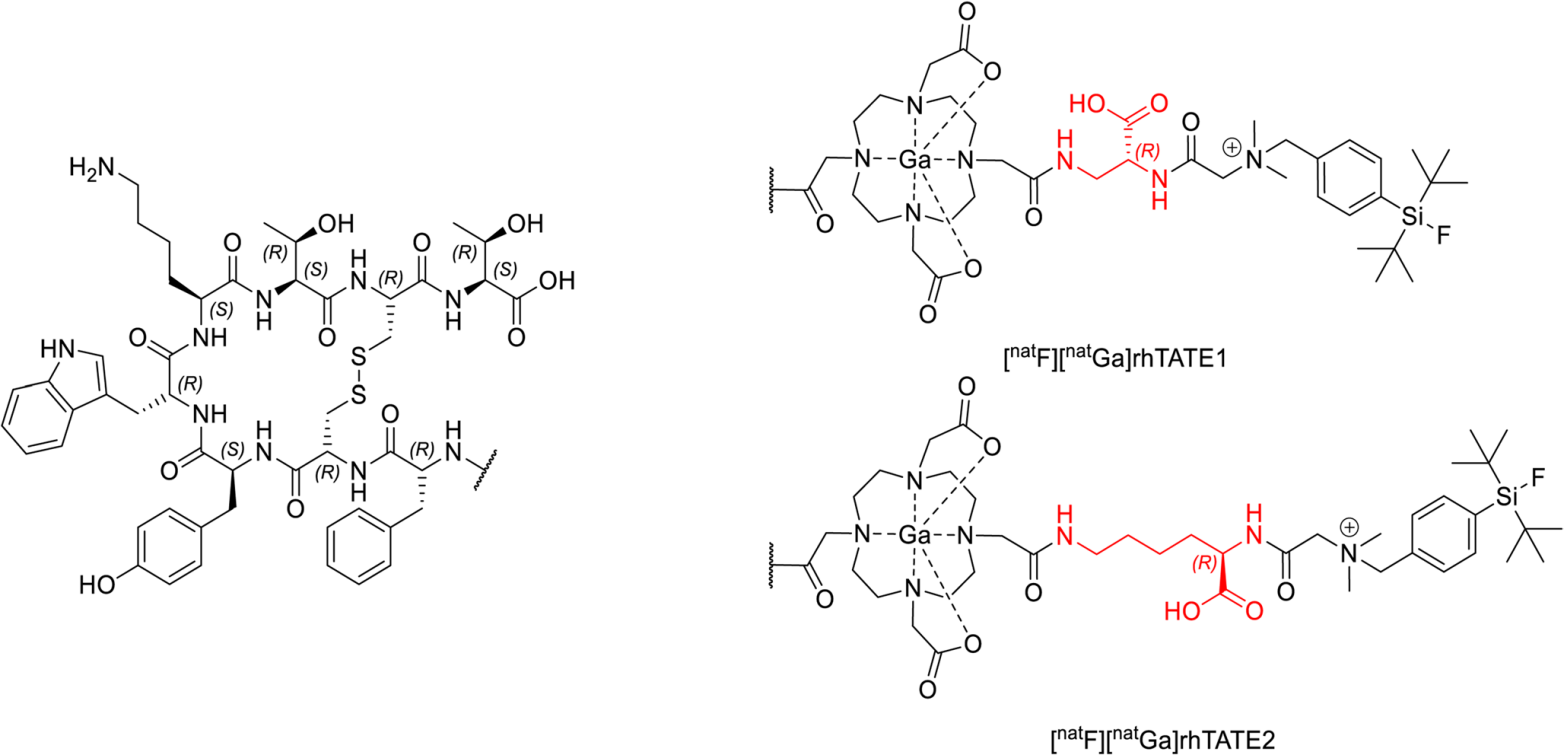
Structures of the first-generation ligands [^nat^F][^nat^Ga]rhTATE1 and [^nat^F][^nat^Ga]rhTATE2. The linkers (rhTATE1: based on d-Dap; rhTATE2: based on d-Lys) are highlighted in red

**Fig. 2:**
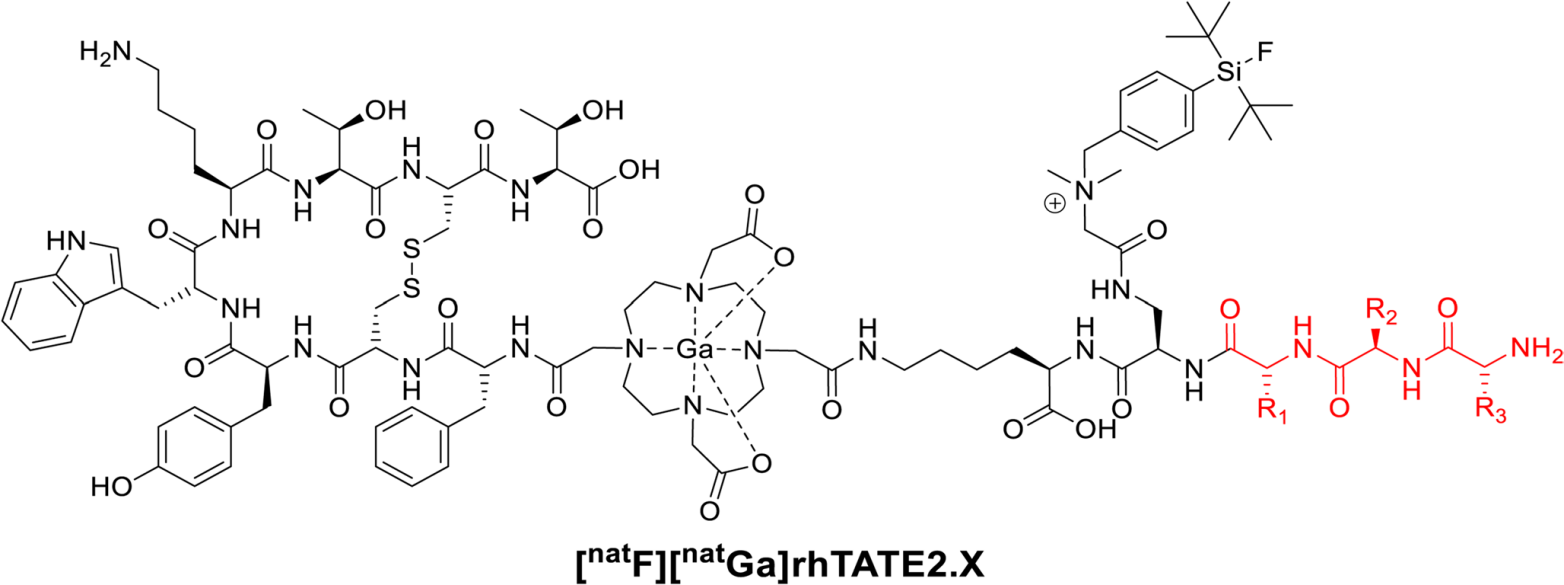
Schematic structures of the second-generation ^nat^Ga^3+^ complexed compounds ([^nat^F][^nat^Ga]rhTATE2.X). The *N*-terminal pharmacokinetic modifiers, based on varying hydrophilic tripeptides are highlighted in red

A key limitation of ^18^F-based PET imaging is the need for a nearby cyclotron. The radiohybrid (rh) concept, successfully applied to prostate specific membrane antigen (PSMA)-targeted tracers [14–16], enables labeling corresponding precursors with ^18^F and ^68^Ga for diagnostic imaging and with ^177^Lu (and ^90^Y) for radioligand therapy. This is achieved by incorporating both a DOTA chelator and a SiFA-moiety into a single molecule. Labeling the ^nat^Ga-DOTA precursor with ^18^F or the free DOTA precursor with ^68^Ga, produces molecular twins with identical biological properties, allowing flexible PET radionuclide choice. To apply this concept for SST_2_-targeted imaging of NETs, we adapted it for compatibility with the TATE targeting vector by designing a compact molecule where DOTA is integrated as a bridging unit between TATE and the SiFA*lin*-moiety (Fig. 1) [17]. Pharmacokinetics were further optimized by *N*-terminal extension with hydrophilic tripeptides (Fig. 2). All compounds were extensively evaluated in vitro and in vivo against the clinical benchmark [^18^F]SiTATE [11, 18].

## Methods

### Synthesis

Detailed synthetic procedures, including reagent equivalents, specific reagents, reaction times, and yields are given in the supplementary materials. In short, the TATE-binding motif was synthesized via solid phase peptide synthesis (SPPS) and cyclized on-resin [11, 19] (Supplementary Fig. 1). DOTA(*t*Bu)_2_ was coupled [17] followed by spacers, hydrophilic modifiers and dimethylglycine (Supplementary Fig. 2). Branching was introduced by the addition of Fmoc-d-Dap(Dde)-OH followed by Dde-deprotection. Finally, SiFA*lin* was formed from the dimethylglycine residue using SiFA-Br (Supplementary Fig. 3). The compounds were cleaved from the resin, purified, and complexed with ^nat^Ga^3+^ or radiolabeled using ^68^Ga^3+^. The synthetic pathways, structures, reversed-phase high performance liquid chromatography (RP-HPLC), and electrospray ionization mass spectrometry (ESI-MS^+^) data are given in the supplementary material.

RP-HPLC was performed using a Shimadzu Corp. HPLC system (Kyoto, Japan) and a reversed-phase column (analytical: MultoKrom^®^ 100-5 C18 (5 μm, 125 × 4.6 mm), preparatory: multospher 100 C18 (5 μm, 250 × 20 mm); CS GmbH, Langerwehe, Germany) with a linear gradient of acetonitrile (ACN) in H_2_O with 0.1% trifluoroacetic acid (TFA).

### Radiolabeling

#### ^18^F fluorination

^18^F fluorination by ion exchange was performed using a modified “*Munich method*” [20]. The desired amount of ^18^F-fluoride was loaded onto a QMA Cartridge (Sep-Pak Accell Plus QMA Plus Light Cartridge, 130 mg sorbent/cartridge, 37–55 μm, preconditioned: 5 ml MeOH and 5 ml H_2_O). Dry dimethylsulfoxide (DMSO; 8 ml) was passed through the cartridge followed by air. The purified ^18^F^−^ was eluted from the cartridge (500 µl NH_4_OAc, 1 m in anhydrous DMSO). The labeling precursor (30 µl, 1mm in DMSO), was added to the ^18^F-fluoride containing cartridge eluate and left to react for 5 min at room temperature (RT). 10 ml phosphate buffer (pH = 3) was added, the mixture loaded onto an HLB cartridge (Oasis^®^ HLB 6 cc Vac cartridge; 30 mg sorbent, preconditioned: 5 ml MeOH and 5 ml H_2_O), the cartridge washed with 10 ml PBS (pH = 7.4), and residual solvent removed using air. The radioactive product was eluted with 300 µl EtOH/PBS (7/3, *v/v*).

#### Automated ^18^F fluorination

Radiolabeling was performed using a Scintomics GRP 3 V module (Fürstenfeldbruck, Germany) with a two valve unit setup (Supplementary Fig. 4) based on the radiosynthesis of [^18^F]SiTATE [21]. ^18^F was dried using a QMA cartridge (preconditioned: 5 ml H_2_O, washed with 5 ml anhydrous MeCN, eluted with 40 mg NH_4_HCOO in 600 µl DMSO), combined with the precursor (1 mm in anhydrous DMSO) and left to react (5 min, RT). Purification was performed using a C18 Plus cartridge (preconditioned: 5 ml EtOH and 5 ml H_2_O), the product eluted in EtOH/H_2_O (50% *v/v*) and sterile filtered using a Cathivex^®^-GV filter (Merck, Darmstadt, Germany). Analysis was performed by radio RP-HPLC (Supplementary Fig. 5).

#### ^68^Ga labeling

^68^Ga was eluted (3 ml, 0.1 m HCl) from a ^68^Ge/^68^Ga generator (Eckert & Ziegler GalliaPharm™, Berlin) directly into a solution of the precursor (10 µl, 10 nmol) in 2-[4-(2-hydroxyethyl)piperazin-1-yl]ethanesulfonic acid (HEPES) buffer (1 ml; 1 g/ml in H_2_O) and left to react (5 min, 60 °C). The reaction mixture was loaded onto a C-18 cartridge (preconditioned: 5 ml MeOH and 5 ml 0.1 m citrate buffer, pH = 5.0), washed with 10 ml citrate buffer and eluted using MeCN with 0.1% TFA. Quality control was performed by radio-thin layer chromatography (TLC; Citrate buffer: Citric acid/NaOH, 0.1 m, pH = 5.0; R_f_: Product: 0.0–0.2.0.2; ^68^Ga: 0.8–1.0.8.0) and radio-RP-HPLC (10–90% ACN/H_2_O with 0.1% TFA *v/v*, 15 min; t_R_ = 10.8 min, K’ = 4.4).

#### Automated ^68^Ga labeling

Radiolabeling was performed using a Scintomics GRP 3 module with a two-valve unit setup (Supplementary Fig. 6). ^68^Ga^3+^ cations are trapped on a PS-H^+^ cartridge (Chromafix, Macherey-Nagel, Düren, Germany) and eluted (2.1 ml; 3.33 m NaCl in 0.71 m HCl). The eluate is combined with the precursor (10–150 nmol) and an aqueous HEPES solution (1.5 ml, 1.5 m). The mixture is left to react (10 min, 70 °C) and purified using a C-18 cartridge (preconditioning: 5 ml H_2_O, 5 ml 0.1 m citric acid buffer (pH = 5.0), washing: 5 ml 0.1 m citric acid buffer (pH = 5.0), elution: EtOH/H_2_O (50% *v/v*)).

#### Radioiodination of TOC

Radioiodination of Tyr^3^-octreotide (TOC) was performed using the Iodogen method [22]. TOC (0.1 mg) was dissolved in anhydrous DMSO (20 µl) and added to 280 µl TRIS iodination buffer (25 mm Tris-HCl, 0.4 m NaCl, pH = 7.5). The mixture was transferred to a 1.5 ml reaction vessel coated with 150 µg of IodoGen^®^, and [^125^I]NaI (5–10 µl, 15–18 MBq, in 0.1m NaOH; Hartmann Analytics, Braunschweig) was added to the mixture and left to react (15 min, RT). The crude product was purified by RP-HPLC (ACN/H_2_O: 10–60% with 0.1% TFA in 15 min; *v/v*) and diluted using assay medium (0.1 nm based on a specific activity at the time point of radiolabeling of 2000 Ci/mmol).

### HSA binding studies

For the determination of binding to human serum albumin (HSA) using HPLC, a Chiralpak HSA column (50 × 3 mm, 5 μm, H13H-2433) was used at a constant flow rate of 0.5 ml/min. The mobile phase (A: NH_4_OAc, 50 mm in H_2_O, pH 6.9 and B: iPrOH) was freshly prepared for each day.

### Lipophilicity (LogD_pH=7.4_)

The LogD_pH=7.4_ was determined using a modified shake flask method as previously described [23].

### Determination of IC_50_

CHO cells stably transfected with SST_2_ (Dr. Jenny Koenig, Cambridge, UK) were grown in DMEM/F12 (with 10% fetal calf serum (FCS); 37 °C; 5% CO_2_) and seeded in 24-well plates 24 ± 2 h before the experiment (1.0 × 10^5^ cells in 1 ml/well medium). After removal of the culture medium, cells were washed with 400 µl of Hank’s Balanced Salt Solution with 1% bovine serum albumin (HBSA) and fresh HBSA (200 µl) was added. Either 25 µl HBSA (control) or the respective ligand in increasing concentrations (10^−10^ – 10^−4^ m in HBSA) were added with subsequent addition of 25 µl of [^125^I]TOC (1.0 nm in HBSA) per well. After incubation (60 min, RT), the assay medium was collected, the cells washed with cold PBS (300 µl), and the fractions combined (unbound activity). The cells were lysed with 1 m NaOH (300 µl, 15 min, RT) and combined with 300 µl 1 m NaOH for rinsing. The activities of the cell-bound and unbound fractions were quantified (γ-counter). Investigations occurred on three separate days (*n* = 3 each), and mathematical analyses was carried out using the GraphPad PRISM software.

### Internalization studies

AR42J cells (European Collection of Cell Cultures, Salisbury, UK) were grown in RPMI 1640 medium (with 2 mm, l-Glu, 10% FCS; *v/v*, 37 °C, 5% CO_2_), harvested 24 ± 2 h and seeded in 24-well poly-l-lysine plates (2.0 × 10^5^ cells/well in 1 ml culture medium). After removal of the medium, the cells were washed with assay medium (300 µl, RPMI 1640 with 2 mm l-Glu, 5% BSA; *v/v*) and incubated in assay medium (200 µl, 15 min, 37 °C, 5% CO_2_). 25 µl of the ^18^F-labeled ligand (20 nm) and [^125^I]TOC (1 nm) in assay medium were added followed by either 25 µl TOC in assay medium (100 µm, competition experiment) or 25 µl of assay medium (internalization experiment). Cells were incubated at 37 °C for 15, 30, and 60 min (*n* = 3 per compound and time point), respectively, and chilled on ice. The supernatant was collected, the well washed with ice-cold RPMI 1640 (300 µl), and the two fractions combined (unbound ligand). The cells were incubated with acid wash solution (300 µl, 0.9% NaCl, 50 mm NaOAc/AcOH buffer, pH = 4.6, 15 min, 4 °C) to remove receptor-bound radioligand. The supernatant was collected, and the cells washed with 300 µl ice-cold acid wash solution (combined fractions: acid releasable, receptor bound ligand). The cells were lysed with NaOH solution (300 µl, 1 m, 15 min, RT), washed with 300 µl of the same solution, the two fractions combined (internalized ligand), and all activities quantified using a γ-counter.

### Animal studies

The 8-week-old female CD1 nu/nu mice were purchased from Charles River (Sulzfeld, Germany). Animal experiments were conducted according to general animal welfare regulations (German animal protection act, as amended on 18.05.2018, Art. 141 G v. 29.3.2017 I 626, approval no. 55.2-1.2-54–2532-71-13 by the General Administration of the Free State of Bavaria; Swiss animal welfare regulations: Animal Welfare Act SR 455, license: Canton de Vaud, 3781 d). To establish tumor xenografts, AR42J cells (5 × 10^6^ cells in 100 µl serum-free culture medium) were inoculated subcutaneously onto the shoulder. Mice were used for experiments when tumors had grown to a diameter of 5–9 mm (7–15 days after inoculation).

#### Biodistribution studies

Approximately 0.5–2.5 MBq (0.05–0.20 nmol) of the ^18^F-labeled ligands were injected into the tail vein of AR42J xenograft-bearing mice under isoflurane anesthesia (1.5% alveolar concentration). The mice were sacrificed 1 h post injection (*n* = 3–5). Organs were removed, weighed, and the activity quantified (γ-counter).

#### µPET imaging

4–5 MBq (0.3–0.5 nmol) of the ^68^Ga-labeled SST_2_-ligand were injected into the tail vein of the xenograft-bearing mice. After 1 h, the mice were anesthetized (isoflurane: 1.5% alveolar concentration) and a PET image acquired (20 min, 32 × 32, 0.5 mm) using an Albira PET/SPECT/CT (Bruker Biospin Corporation, Woodbridge, CT, USA) followed by a CT scan (10 min). For blocking, 20 µg octreotide (0.8 mg/kg) were co-injected with the tracer. The images were reconstructed (Albira reconstructor, version NMI3.3) and analyzed (PMOD, V6.3.4, Bruker).

### In-vitro stability assay

[^nat^F][^68^Ga]rhTATE (ca. 5 MBq) was added to 500 µl samples of mouse liver extract, human serum, or PBS, respectively. The mixtures were incubated at 37 °C for 60 min. Workup of blood and liver extract samples was performed using cartridge purification (StrataX cartridge, 33 μm Polymeric Reversed Phase, 500 mg; preconditioning: 500 µl MeOH, 5 ml H_2_O; washing: 3 ml H_2_O; elution: ACN/H_2_O (0.1% TFA, 1:3, *v/v*, 300 µl). The resulting eluates were analyzed using radio RP-HPLC (Chromolith^R^ RP-18e; 100 × 4.6 mm; *Merck KGaA*; gradient of 10–90% ACN in H_2_O (0.1% TFA) within 8 min; flow rate: 1 ml/min). For cross-validation of HPLC results, radio RP-TLC was equally performed for all samples (1 m citric acid/NaOH, pH = 5.0; Rf: Product: 0.0–0.2.0.2; ^68^Ga: 0.8–1.0.8.0).

### Statistical analysis

An unpaired Welch’s t-test was applied using the GraphPad Prism software (version 10.1.2). Standard deviations for tumor-to-organ (T/O) ratios were calculated using the rules of error propagation. Thresholds for statistical significance are defined as: *p* > 0.05 (ns); *p* ≤ 0.05 (*); *p* ≤ 0.01 (**); *p* ≤ 0.001 (***); *p* ≤ 0.0001 (****).

## Results

### First-generation ligands

First-generation compounds (Fig. 1) were synthesized to investigate the feasibility of generating rh SST_2_ ligand based on the use of the *trans*-DOTA-chelator as a bridging unit. Two radiohybrid TATE derivatives (rhTATE) with varying spacer lengths were synthesized, using d-Dap (rhTATE1) and d-Lys (rhTATE2).

The ^nat^Ga^3+^-complexes, [^nat^F][^nat^Ga]rhTATE1 (*N*-SiFA*lin*-*N*,* N*-Me_2_-Gly-d-Dap(*trans*-[^nat^Ga]DOTA-TATE) and [^nat^F][^nat^Ga]rhTATE2 (*N*-SiFA*lin*-*N*,* N*-Me_2_-Gly-d-Lys(*trans*-[^nat^Ga]DOTA-TATE), were investigated in vitro regarding their affinity to SST_2_, hydrophilicity, and binding to HSA (Table 1).

**Table 1:** IC_50_ values were determined using CHO cells stably overexpressing hSST_2_ and [^125^I]TOC as the radioligand. Data represent means from 3 separate determinations (mean ± standard deviation (SD)) with *n* = 3, respectively. LogD_pH=7.4_ of the radioligands between PBS (pH = 7.4) and n-octanol were determined using a shake-flask method [23] and represent mean of *n* = 8. Affinity to HSA was determined using high performance affinity chromatography (HPAC)

Name	Linker	IC_50_ [nm]	LogD_pH=7.4_	HSA HPAC %
[^nat/18^F][^nat^Ga]rhTATE1	d-Dap	5.6 ± 1.4	−1.03	95.4
[^nat/18^F][^nat^Ga]rhTATE2	d-Lys	5.7 ± 0.2	−1.19	94.5
[^nat/68^Ga]DOTA-TATE	-	1.9 ± 0.1	−3.69 [24]	88.9
[^nat/18^F]SiTATE	-	7.0 ± 0.6	−1.27	86.8

Both compounds showed high SST_2_ affinity, which was slightly enhanced compared to SiTATE, but significantly lower compared to [^nat^Ga]DOTA-TATE. Compared to [^nat/18^F]SiTATE, both [^nat/18^F][^nat^Ga]rhTATE1 and [^nat/18^F][^nat^Ga]rhTATE2 showed higher lipophilicities and enhanced plasma protein binding.

### Second-generation ligands

The second-generation analogs (H-AA3-AA2-AA1-D-Dap(*N*-SiFA*lin*-*N*,*N*-Me_2_-Gly)-D-Lys(*trans*-DOTA-TATE)-OH) were derived from the more hydrophilic [^18/nat^F][^68/nat^Ga]rhTATE2. For the simultaneous integration the SiFA*lin*-moiety and hydrophilic modifiers, an additional branching unit (d-Dap) was introduced to accommodate SiFA*lin* in the sidechain. The structures of the second-generation analogs are schematically shown in Fig. 2.

The different hydrophilic modifiers are composed of three hydrophilic amino acids with varying charge and sidechain lengths (Table 2).

**Table 2:** Amino acid sequences of the hydrophilic modifiers of the second-generation compounds. Numbers in brackets indicate the net charge of the respective amino acid as integrated into the sequence at physiological pH

Compound	AA1	AA2	AA3	Peptide net charge
rhTATE2.1/[^nat^Ga]rhTATE2.1	d-Cit (0)	d-Cit (0)	d-Cit (0)	+ 2
rhTATE2.2/[^nat^Ga]rhTATE2.2	d-Cit (0)	d-Cit (0)	d-Glu (−1)	+ 1
rhTATE2.3/[^nat^Ga]rhTATE2.3	d-Dap (+ 1)	d-Glu (−1)	d-Glu (−1)	+ 1
rhTATE2.4/[^nat^Ga]rhTATE2.4	d-Lys (+ 1)	d-Glu (−1)	d-Glu (−1)	+ 1
rhTATE2.5/[^nat^Ga]rhTATE2.5	d-Glu (−1)	d-Glu (−1)	d-Glu (−1)	−1

### In vitro evaluation

The respective ^nat^Ga^3+^-complexes of rhTATE2.1–5, namely H-AA3-AA2-AA1-d-Dap(*N*-SiFA*lin*-*N*,*N*-Me_2_-Gly)-d-Lys(*trans*-[^nat^Ga]DOTA-TATE)-OH, were evaluated in vitro with respect to affinity (IC_50_), hydrophilicity (LogD_pH=7.4_), HSA binding and internalization (normalized to internal reference [^125^I]TOC) (Table 3).

**Table 3:** IC_50_ values were determined using CHO cells stably overexpressing hSST_2_ and [^125^I]TOC as radioligand. Data represent means ± SD from 3 separate determinations with *n* = 3, respectively. LogD_pH=7.4_ of the radioligands between PBS (pH = 7.4) and n-octanol were determined using a shake-flask method [23] and represent mean of *n* = 8. Affinity to HSA was determined using affinity chromatography. Internalization of the ^18^F-labeled compounds into AR42J cells and data are given as a percentage compared to the internal reference [^125^I]TOC

Compound	IC_50_ [nm]	LogD_pH=7.4_	HSA HPAC [%]	Internalization [%]
[^18/nat^F][^nat^Ga]rhTATE2.1	3.7 ± 0.5	−2.27	98.8	536
[^18/nat^F][^nat^Ga]rhTATE2.2	3.3 ± 0.5	−2.30	94.3	550
[^18/nat^F][^nat^Ga]rhTATE2.3	2.6 ± 0.2	−2.12	96.0	619
[^18/nat^F][^nat^Ga]rhTATE2.4	3.2 ± 0.6	−2.28	92.7	841
[^18/nat^F][^nat^Ga]rhTATE2.5	2.8 ± 0.2	−2.30	84.8	357
[^18/nat^F][^nat^Ga]rhTATE2	5.7 ± 0.2	−1.19	94.5	-
[^nat/68^Ga]DOTA-TATE	1.9 ± 0.1	−3.69 [24]	88.9	570
[^nat/18^F]SiTATE	7.0 ± 0.6	−1.27	86.8	236

Compared to the parent compound [^nat^F][^nat^Ga]rhTATE2 and the reference SiTATE, all five novel derivatives show up to twofold enhanced SST_2_ affinity. Their hydrophilicity is enhanced by approximately one order of magnitude relative to [^nat^F][^nat^Ga]rhTATE2 and are substantially more hydrophilic than [^18^F]SiTATE. Interestingly, the compounds vary in HSA binding: [^nat^F][^nat^Ga]rhTATE2.2–2.4.4 display similar binding to [^nat^F][^nat^Ga]rhTATE2, [^nat^F][^nat^Ga]rhTATE2.1 demonstrates exceptionally high HSA binding, while [^nat^F][^nat^Ga]rhTATE2.5 closely matches the reference [^18^F]SiTATE.

Comparative internalization studies using AR42J carcinoma cells included [^125^I]TOC as an internal reference to control for inter-experimental variation. All compounds show increased internalization compared to [^18^F]SiTATE. [^18^F][^nat^Ga]rhTATE2.1–3 are internalized similarly to [^68^Ga]DOTA-TATE, while[^18^F][^nat^Ga]rhTATE2.5 shows slightly lower, yet still improved, internalization over [^18^F]SiTATE. The highest internalization was observed for [^18^F][^nat^Ga]rhTATE2.4.

To assess the impact of Ga-complexation on affinity, the “free DOTA” ^18^F-labeled compounds were also evaluated (Supplementary Table 1). All showed 2- to 4-fold lower affinities compared to their Ga-complexed counterparts. Hydrophilicity remained largely unchanged, while HSA binding generally decreased, except for [^nat^F]rhTATE2.5, which matched the binding of [^nat^F][^nat^Ga]rhTATE2.5.

These results demonstrate that the second-generation ^nat^Ga complexes are suitable for in vivo studies. [^18^F][^nat^Ga]rhTATE2.2 and [^18^F][^nat^Ga]rhTATE2.5, in particular, exhibit high affinity, identical hydrophilicity, and similar internalization kinetics. To assess the impact of their differing HSA binding on the in vivo performance, these compounds were selected for in vivo investigations, with [^18^F]SiTATE as a reference.

### In vivo evaluation

**Fig. 3:**
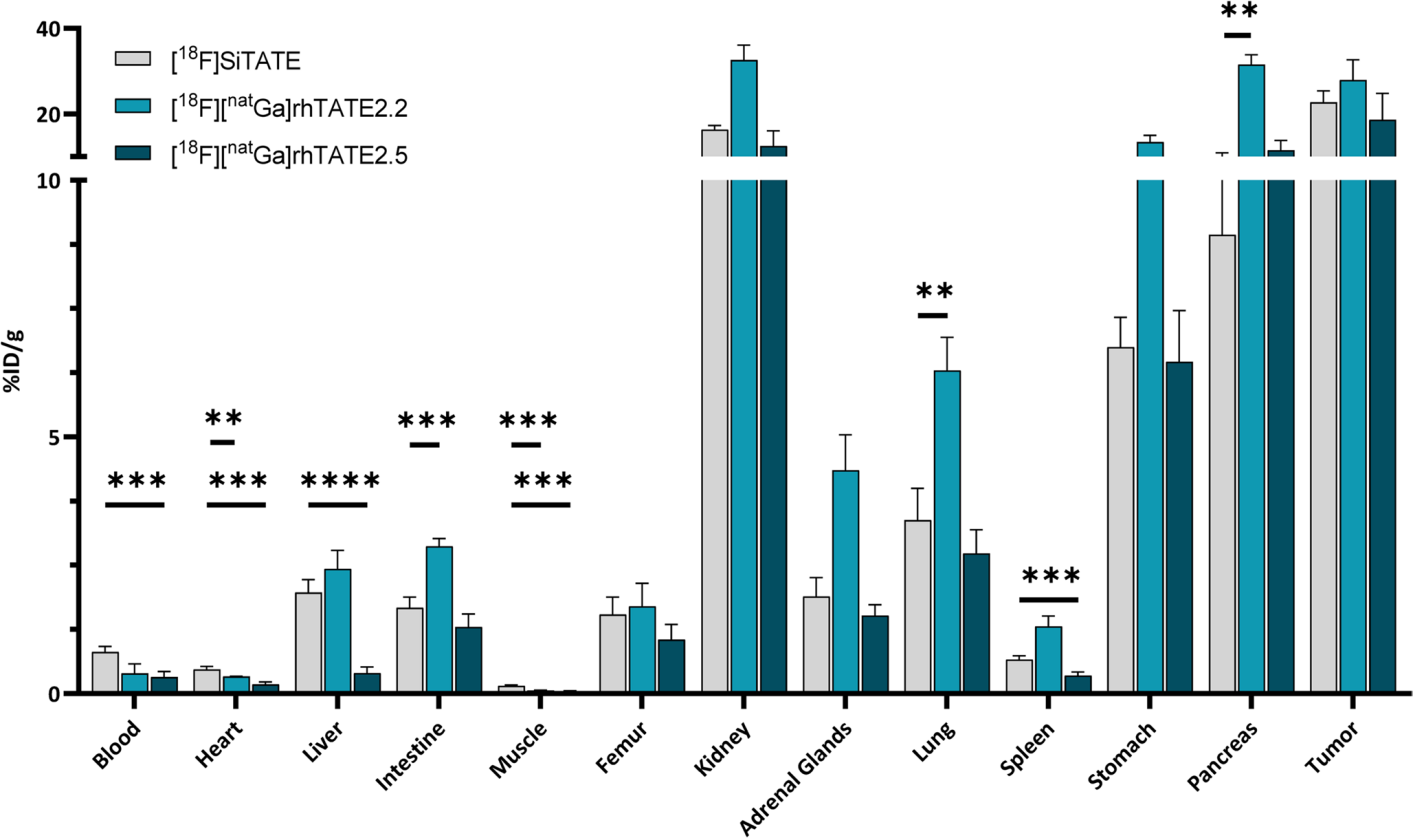
Biodistribution of [^18^F][^nat^Ga]rhTATE2.2, [^18^F][^nat^Ga]rhTATE2.5 and [^18^F]SiTATE at 1 h p.i. in AR42J tumor-bearing female CD1 nu/nu mice (*n* = 3 for [^18^F][^nat^Ga]rhTATE2.2, *n* = 4 for [^18^F][^nat^Ga]rhTATE2.5, *n* = 5 for SiTATE). Values are expressed as a percentage of injected dose per gram (%ID/g), mean ± SD. Statistical significance is calculated compared to [^18^F]SiTATE

The results of the biodistribution study in AR42J tumor bearing nude mice at 1 h p.i. are summarized in Fig. 3 (explicit numerical values: Supplementary Table 2).

Both novel compounds demonstrate enhanced blood clearance compared to [^18^F]SiTATE. [^18^F][^nat^Ga]rhTATE2.5, with significantly lower HSA binding compared to [^18^F][^nat^Ga]rhTATE2.2 (Table 2) despite identical logD, shows substantially reduced background uptake in excretion organs (liver, intestines, kidney). However, faster excretion also leads to a lower uptake in organs with high endogenous SST_2_ expression (pancreas, stomach, kidney, adrenal glands). This trend is also observed in the tumor but is less pronounced. Overall, [^18^F][^nat^Ga]rhTATE2.5 exhibits a biodistribution similar to [^18^F]SiTATE but with markedly reduced hepatobiliary clearance due to lower lipophilicity (Table 3).

**Fig. 4:**
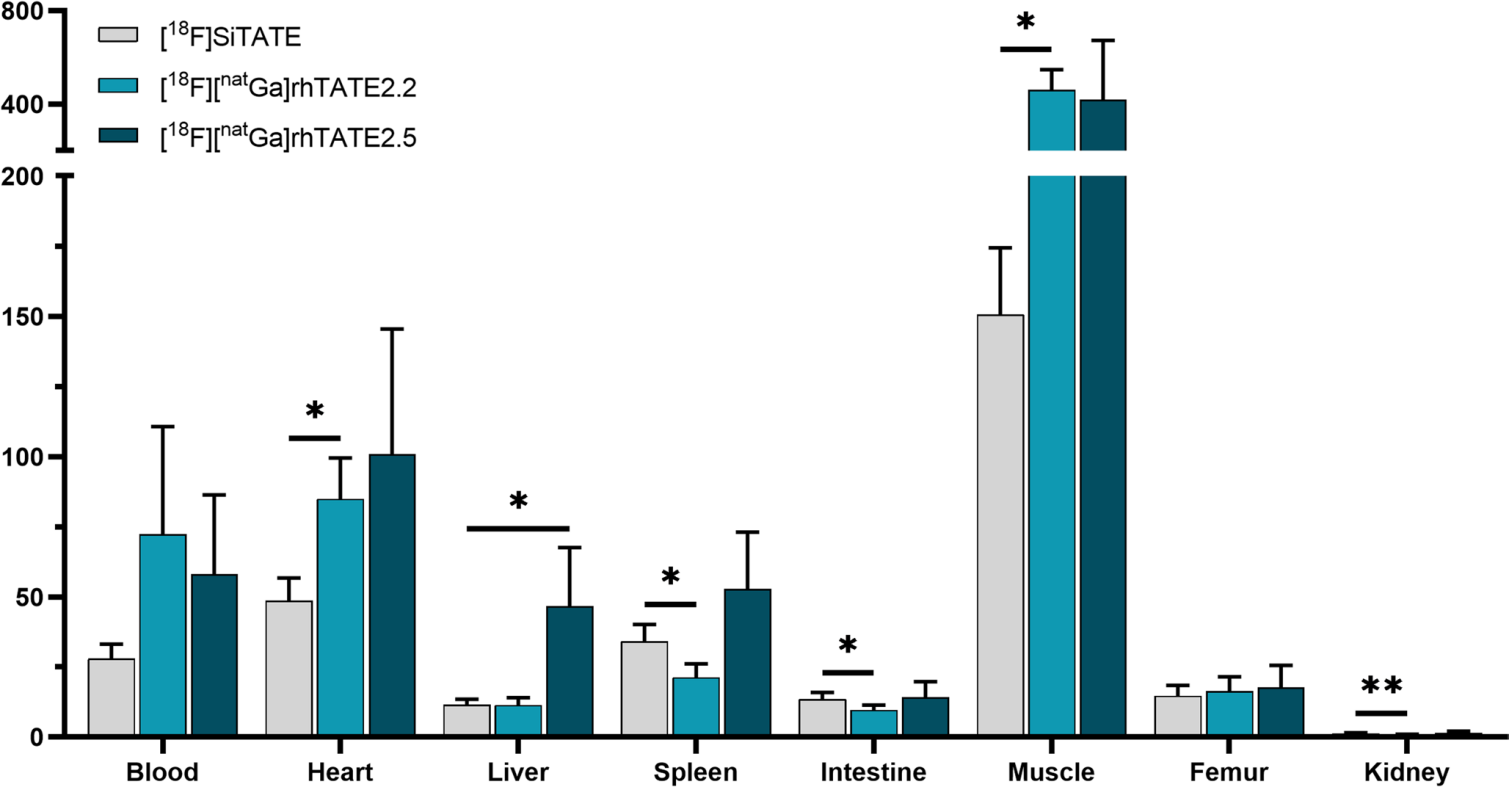
T/O ratios of [^18^F]SiTATE, [^18^F][^nat^Ga]rhTATE2.2, and [^18^F][^nat^Ga]rhTATE2.5 (0.05–0.2 nmol, 0.5–2.5 MBq) at 1 h p.i. in relevant organs of AR42J tumor-bearing female CD1 nu/nu mice (*n* = 3 for [^18^F][^nat^Ga]rhTATE2.2, *n* = 4 for [^18^F][^nat^Ga]rhTATE2.5, *n* = 5 for SiTATE). Values represent mean ± SD. Statistical significance is calculated compared to [^18^F]SiTATE

Figure 4 summarizes the T/O ratios for [^18^F][^nat^Ga]rhTATE2.2, [^18^F][^nat^Ga]rhTATE2.5, and [^18^F]SiTATE (explicit numerical values for all tissue: Supplementary Table 3). Owing to either high tumor uptake ([^18^F][^nat^Ga]rhTATE2.2) or low background accumulation ([^18^F][^nat^Ga]rhTATE2.5), both novel rh compounds display significantly improved T/O ratios compared to [^18^F]SiTATE.

**Fig. 5:**
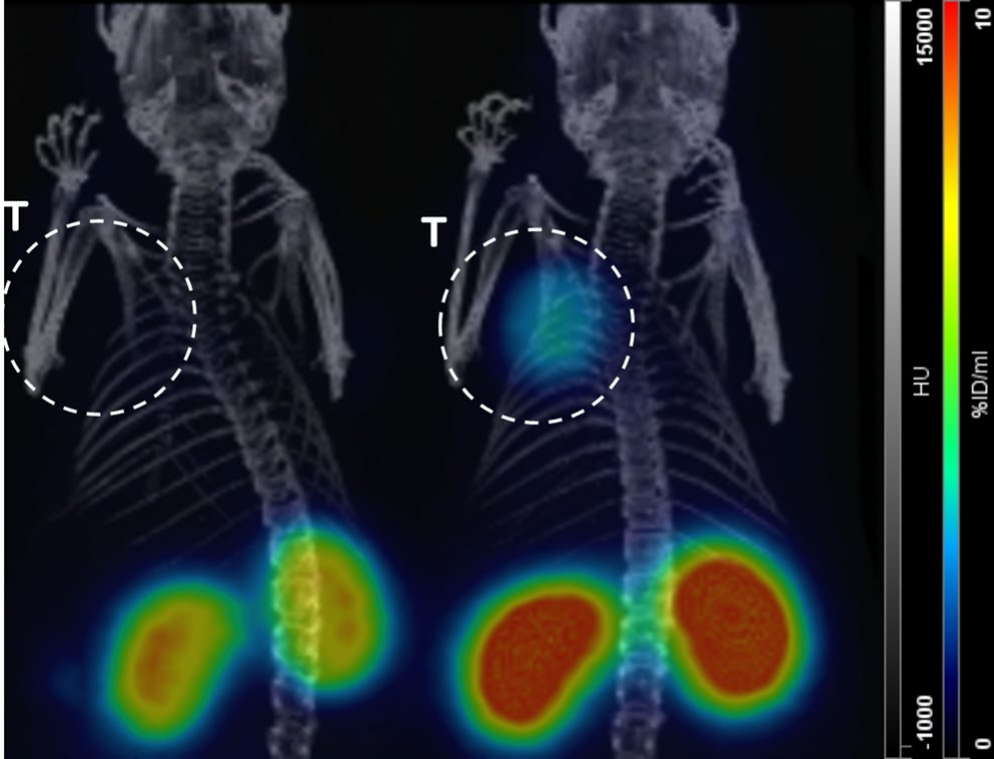
MIP µPET images of AR42J tumor-bearing female CD1 nu/nu mice (60 min p.i.) of [^nat^F][^68^Ga]rhTATE2.5 (4 MBq, 0.5 nmol tracer/mouse, left mouse: co-injection with 20 µg octreotide)

To corroborate these results and demonstrate the SST_2_ specificity of [^nat^F][^68^Ga]rhTATE2.5, µPET/CT imaging of AR42J tumor bearing CD1 nu/nu mice (control vs. blocking with octreotide (20 µg octreotide/mouse; 0.8 mg/kg)) was performed (Fig. 5).

As anticipated from the biodistribution study, µPET images demonstrate intense [^nat^F][^68^Ga]rhTATE2.5 uptake in the tumor and kidneys of the control mouse (right). In contrast, the mouse co-injected with 20 µg octreotide (left) exhibits no detectable tumor signal and significantly reduced kidney activity due to SST_2_ blocking.

### Automated ^18^F-radiolabeling

To enable rapid clinical translation, an automated good manufacturing practice (GMP)-compliant ^18^F-labeling protocol for [^18^F][^nat^Ga]rhTATE2.5 was established. Starting from 150 nmol precursor, the tracer was synthesized in 21 min with a 62.2% decay-corrected radiochemical yield, > 99.5% radiochemical purity (Supplementary Fig. 5), and a molar activity of 7.35 GBq/µmol (starting from 2.11 GBq [^18^F]fluoride). Gas chromatography confirmed the final DMSO concentration (0.058 mg/ml) was below the permissible limit (0.25 mg/ml).

### In vitro complex stability investigation

To ensure the stability of the ^68^Ga-trans-DOTA complex in [^nat^F][^68^Ga]rhTATE2.5 under physiological conditions, the in vitro stability of [^nat^F][^68^Ga]rhTATE2.5 was assessed in PBS, human serum, and mouse liver extract (1 h, 37 °C; Supplementary Fig. 7). [^nat^F][^68^Ga]rhTATE2.5 was found to be stable over 60 min under all investigated conditions. Minor side peaks observed in the original tracer preparation (Supplementary Fig. 7; total of < 15% of the area under the curve (AUC)) were also present in the incubated samples, with no significant changes in the relative AUC proportions, indicating high in vitro stability of [^nat^F][^68^Ga]rhTATE2.5.

## Discussion

Extensive literature shows that *N*-terminal modifications of SST_2_-targeted peptides (e.g. TOC, TATE, JR11, LM3) greatly affect receptor affinity. Even radiometal exchange in the *N*-terminal chelator (DOTA or NOTA) can impact the affinity by tenfold [24, 25]. Additionally, the linker structure between the peptide core and chelator has substantial influence on ligand affinity, internalization and cellular retention [26]. It was therefore important to assess whether the TATE scaffold tolerates the *N*-terminal modifications needed for a SiFA/DOTA-based rh concept. Building on the observation that direct attachment of a Ga-DOTA-complex to TATE (as in [^68^Ga]DOTA-TATE) entails excellent SST_2_-affinity [24], DOTA was directly conjugated to TATE and used as a bridge to the SiFA*lin*-moiety, maintaining a compact tracer design [17].

The first-generation compounds, [^18/nat^F][^nat/68^Ga]rhTATE1 and [^18/nat^F][^nat/68^Ga]rhTATE2 demonstrate that the *trans*-DOTA-based rh ligand design is both chemically accessible [17] and yields ligands with high SST_2_ affinity. Although Ga-chelation was slightly slower compared to mono-conjugated DOTA, complexation remained efficient since the Ga^3+^ ion forms a hexacoordinated complex with DOTA, with only two carboxylate pendant arms involved in Ga^3+^ coordination [27]. As observed in first-generation SiFA- and SiFA*lin*-radiopeptides [10], the SiFA*lin* moiety significantly increases tracer lipophilicity. This precludes further in vivo evaluations since TOC- and TATE-based SST_2_-targeted ligands with a LogD > -1.5 generally exhibit non-negligible hepatobiliary excretion. The d-Lys spacer provided slightly improved hydrophilicity (Table 1) and was retained in second-generation ligands.

As summarized in Fig. 2, a d-Dap-residue was introduced as a branching unit to conjugate both the SiFA*lin* moiety and hydrophilic tripeptides as pharmacokinetic modifiers (Table 3). In vitro evaluation of the novel rh analogs [^18/nat^F][^nat/68^Ga]rhTATE2.1–2.5.5 revealed several interesting structure-activity relationships.

First, affinity improved consistently by 1.5- to 2-fold across all compounds, regardless of *N*-terminal tripeptide sequence. This indicates that the novel linker geometry globally enhances ligand-receptor interaction, emphasizing the importance of linker selection for optimizing affinity in radiolabeled octreotide/TATE-based tracers [26]. Affinity data for the metal-free DOTA precursors (Supplementary Table 1), showing a 3- to 4-fold reduction, confirm that direct *N*-terminal conjugation of TATE with *trans*-[^nat^Ga]DOTA benefits binding affinity.

Second, second-generation rh analogs show substantially enhanced internalization compared to [^18^F]SiTATE (Table 3). This correlates with the improved affinity but is also influenced by the pharmacokinetic modifier’s amino acid composition. Analogs with primarily uncharged d-Cit residues internalize similarly to [^68^Ga]DOTA-TATE, while the presence of both negative and positive charges in [^18^F][^nat^Ga]rhTATE2.3 and [^18^F][^nat^Ga]rhTATE2.4 seems to enhance internalization. The all-d-Glu tripeptide in [^18^F][^nat^Ga]rhTATE2.5 reduces internalization. However, this remains speculative as affinity- and charge-independent effects on SST_2_ internalization have been reported [28–30].

Third, all second-generation compounds exhibit nearly identical lipophilicities, about one order of magnitude lower than [^18/nat^F][^nat^Ga]rhTATE2 (Table 3). This was independent of the net charge or charge distribution of the pharmacokinetic modifier, though HSA binding tended to vary with the number and type of charges in the *N*-terminal tripeptide. Interestingly, as seen in first-generation compounds, the d-Dap residue decreases hydrophilicity (as compared to d-Lys, Tables 1 and 3), and increases plasma protein binding ([^18^F][^nat^Ga]rhTATE2.3 vs. [^18^F][^nat^Ga]rhTATE2.4).

Despite the complex in vitro effects, comparative in vivo evaluation of [^18^F][^nat^Ga]rhTATE2.2, [^18^F][^nat^Ga]rhTATE2.5 and [^18^F]SiTATE identified lipophilicity, peptide net charge, and internalization efficiency as the major factors influencing the biodistribution.

Both [^18^F][^nat^Ga]rhTATE2.5 and [^18^F]SiTATE have a peptide net charge of −1 (Table 2), display nearly identical HSA binding and similar internalization (Table 3). Thus, the differences in biodistribution (faster blood clearance, substantially decreased hepatobiliary excretion of [^18^F][^nat^Ga]rhTATE2.5 compared to [^18^F]SiTATE; Fig. 3) are mainly due to its tenfold reduced lipophilicity. Despite faster clearance, [^18^F][^nat^Ga]rhTATE2.5 maintained uptake in SST_2_-positive tissues, likely due to enhanced internalization compensating for lower plasma concentrations at 1 h p.i.

Direct comparison of [^18^F][^nat^Ga]rhTATE2.2 (net charge + 1) and [^18^F][^nat^Ga]rhTATE2.5 (net charge − 1) reveals two effects. First, kidney uptake of [^18^F][^nat^Ga]rhTATE2.2 is more than twice as high (32.6 ± 3.5 vs. 12.5 ± 3.5%iD/g), consistent with the charge-dependent renal accumulation of peptide tracers [31–35]. Second, [^18^F][^nat^Ga]rhTATE2.2 shows enhanced uptake in all SST_2_ expressing tissues, especially in organs with low endogenous SST_2_ expression (lung, spleen, intestines; Fig. 3, Supplementary Table 2) and in AR42J xenografts (27.9 ± 4.8 vs. 18.6 ± 6.2%iD/g for [^18^F][^nat^Ga]rhTATE2.5 and 22.7 ± 2.7%iD/g for [^18^F]SiTATE). As their affinities of [^18^F][^nat^Ga]rhTATE2.2 and [^18^F][^nat^Ga]rhTATE2.5 are identical, the enhanced internalization of [^18^F][^nat^Ga]rhTATE2.2 likely explains the higher uptake, supported by prior data linking internalization and tumor accumulation [28, 29]. Notably, [^18^F][^nat^Ga]rhTATE2.2 also exhibits markedly increased pancreatic uptake (31.5 ± 2.3%iD/g) compared to both [^18^F][^nat^Ga]rhTATE2.5 and [^18^F]SiTATE (11.5 ± 2.3 and 8.94 ± 1.98%iD/g, respectively) which may lead to false-positive findings in the pancreas, and effect that has already been documented for [^68^Ga]DOTA-TATE [36].

Overall, tracer diagnostic performance depends on target-to-background ratios, which were clearly superior for [^18^F][^nat^Ga]rhTATE2.5, both compared to [^18^F][^nat^Ga]rhTATE2.2 and [^18^F]SiTATE (Fig. 4, Supplementary Table 3). This was particularly pronounced for the liver, which is the primary metastatic site for NETs [37].

## Conclusion

The development of [^18^F][^nat^Ga]rhTATE2.5 exemplifies the successful implementation of a novel class of rhTATE analogs using [^nat/68^Ga]DOTA as a bridging unit. Our data demonstrate that small changes in linker length or pharmacokinetic modifiers significantly influence key properties such as hydrophilicity, net charge, HSA binding, receptor affinity, and internalization efficiency. [^18^F][^nat^Ga]rhTATE2.5 exhibits superior SST_2_-targeting and in vivo performance compared to the clinical reference [^18^F]SiTATE. Its radiohybrid design allows flexible radiolabeling with ^18^F or ^68^Ga, offering clinical versatility. GMP-compliant, automated radiolabeling for both radionuclides further supports clinical translation on a technical level.

## Supplementary Information

Below is the link to the electronic supplementary material.

Supplementary Material 1 (DOCX.18.7 MB)

## Data Availability

All data generated or analyzed during this study are included in this published article and its supplementary information file. Materials were sourced from publicly accessible suppliers.

## Abbreviations

%ID/g: Percent of the injected dose per gram
ACN: Acetonitrile
AMBF_3_: Aminomethyltrifluoroborates
DMSO: Dimethylsulfoxide
DOTA: 2,2’,2’’,2’’’-(1,4,7,10-Tetraazacyclododecane-1,4,7,10-tetrayl)tetraacetic acid
ESI-MS: Electrospray ionization mass spectrometry
FCS: Fetal calf serum
GMP: Good manufacturing practice
HBSA: Hank’s Balanced Salt Solution with 1% bovine serum albumin
HPAC: High performance affinity chromatography
HPLC: High performance liquid chromatography
HSA: Human serum albumin
NET: Neuroendocrine tumors
NOTA: 1,4,7-Triazacyclononane-1,4,7-triacetic acid
PBS: Phosphate buffered saline
PET: Positron emission tomography
PRRT: Peptide-receptor radiotherapy
PSMA: Prostate specific membrane antigen
rh: Radiohybrid
rhTATE: radiohybrid TATE derivative
RP: Reversed phase
RT: Room temperature
SiFA: Silicon fluoride acceptor
SPECT: Single-photon emission computed tomography
SPPS: Solid-phase peptide synthesis
SST_2_: Somatostatin transmembrane receptor 2
T/O: Tumor-to-organ
TATE: (Tyr^3^)-octreotate
TFA: Trifluoroacetic acid
TLC: Thin layer chromatography
TOC: (Tyr^3^)-octreotide
