# Supplementary material for: Novel radiohybrid PET-Tracers for SST2-targeted imaging of neuroendocrine tumors
Source: Eur J Nucl Med Mol Imaging. 2025 Oct 23;53(4):2518–29. doi: 10.1007/s00259-025-07638-9 (PMC12920295; doi:10.1007/s00259-025-07638-9)
Supplement: Supplementary file 1 — Supplementary Material 1 (DOCX.18.7 MB) [file 259_2025_7638_MOESM1_ESM.docx]

# Electronic Supplementary Material

# Novel radiohybrid PET-Tracers for imaging of neuroendocrine tumors

Lennard Wendlinger^1,2,3^, Mara Parzinger^3^, Alexandra Litvinenko^1,2^, David Viertl^1,2^, Philipp Spatz^4^, Alexander Wurzer^3,4^, Hans-Jürgen Wester^3,4,$^, Margret Schottelius^1,2,$,*^

1. *Translational Radiopharmaceutical Sciences, Centre Hospitalier Universitaire Vaudois, 1005 Lausanne, Switzerland*
2. *AGORA, Pôle de recherche sur le cancer, 1011 Lausanne, Switzerland*
3. *Pharmaceutical Radiochemistry, Technical University of Munich, 85748 Garching, Germany*
4. *Scintomics, Molecular Applied Theranostics Technologies GmbH, 82256 Fürstenfeldbruck, Germany*

^$^ both authors contributed equally

***Corresponding Author:**

Prof. Dr. Margret Schottelius

Phone +41.21.545.1120

Mobile +41.79.556.0143

Email: margret.schottelius@chuv.ch

**Peptide Synthesis and Modification:**

The radiohybrid peptides were assembled by SPPS on 2-CTC resin (initial loading: ca.0.5–0.7 mmol/g). For example, ca. 1 g of 2-CTC resin was first loaded with Fmoc-Thr(tBu)-OH (1.0 eq., 2.25 eq. DIPEA in DMF) to afford a resin-bound Thr residue. Subsequent AAs were coupled stepwise using Fmoc chemistry: each Fmoc-protected AAs (1.5 eq per coupling) was activated with a coupling reagent (1.5 eq. TBTU, 1.5 eq. HOAt) and DIPEA (4.5 eq) in DMF. Coupling reactions were carried out for 2 h with gentle agitation.

**Special residues and branches:**

Cysteine (Acm-protected) residues were incorporated using 2.0 eq Fmoc-Cys(Acm)-OH activated with DIC/Oxyma (2.0 eq each, DIPEA 0.8 eq). Divergent branching was achieved via a d-amino acid (lysine/diaminopropionic acid): Fmoc-d-Lys(Fmoc)-OH or Fmoc-d-Dap(Dde)-OH was coupled in the same manner (1.5 eq AA, 1.5 eq TBTU/HOBt, *sym*-collidine 11.0 eq as base). After removing the Dde protecting group (1.25 g hydroxylamine hydrochloride, 0.92 g imidazole in 5 ml NMP and 1 ml DCM, 4 h, room temperature), *N*,*N*-dimethylglycine was attached (1.5 eq AA, standard coupling conditions). The trans-DOTA(tBu)_2_ chelator was introduced in excess (3.0 eq DOTA(tBu)_2_, pre-activated with HOAt/TBTU, 3.0 eq each, 11.0 eq *sym*-collidine) and allowed to react overnight to ensure complete attachment. To attach the silicon fluoride acceptor moiety, SiFA-Br (3.0 eq. in 2 ml DCM, 6.0 eq. DIPEA), and the suspension was stirred overnight in DCM.

Final cleavage of the fully assembled resin-bound peptide (with all acid-labile protecting groups) was carried out with a TFA cocktail (TFA/H₂O/TIPS, 87.5:10:2.5) for 2 × 45 min. The crude product was filtered off the resin, concentrated in a nitrogen stream, and precipitated in cold Et_2_O. Each peptide conjugate was prepared in 25-40 µmol batches, typically yielding ca. 1-3 mg (ca. 2-5%) of the purified, final product.

**Supplementary figure 1:** Reaction scheme for the on-resin solid-phase peptide synthesis followed by selective deprotection of Acm groups and simultaneous oxidative cyclization.

**Supplementary figure 2:** Reaction scheme for the synthesis of first-generation compounds (rhTATE1: n=1; rhTATE2: n=4).

**Supplementary figure 3:** Reaction scheme for the synthesis of second-generation compounds with hydrophilic modifiers comprised of three amino acids (R_1_-R_3_ derived from d-Cit, d-Dap, d-Lys, and d-Glu according to Table 2).

**rhTATE1:***N*-SiFA*lin*-*N,N*-Me_2_-Gly-d-Dap(*trans*-DOTA-TATE)-OH
**RP-HPLC (analytical):** (10-60% MeCN/H_2_O with 0.1% TFA, *v/v*, 15 min): t*_R_* = 12.6 min; K’ = 5.0.
**RP-HPLC (preparative):** (35-47% MeCN/H_2_O with 0.1% TFA, *v/v*, 20 min): t*_R_* = 17.2 min; K’ = 5.2.
**MS** **(ESI positive):** *m/z* calculated for C_87_H_127_FN_17_O_21_S_2_Si^+^: 1856.86, found: 619.9 [M+3H]^3+^, 929.3 [M+2H]^2+^.

**[^nat^Ga]rhTATE1:***N*-SiFA*lin*-*N,N*-Me_2_-Gly-d-Dap(*trans*-[^nat^Ga]DOTA-TATE)-OH
**RP-HPLC (analytical):** (10-60% MeCN/H_2_O with 0.1% TFA, *v/v*, 15 min): t*_R_* = 13.1 min; K’ = 5.2.
**MS** **(ESI positive):** *m/z* calculated for C_87_H_125_FGaN_17_O_21_S_2_Si^+^: 1923.77, found: 642.2 [M+3H]^3+^, 962.7 [M+2H]^2+^, 1283.5 [2M+3H]^3+^.

**rhTATE2:***N*-SiFA*lin*-*N,N*-Me_2_-Gly-d-Lys(*trans*-DOTA-TATE)-OH
**RP-HPLC (analytical):** (10-60% MeCN/H_2_O with 0.1% TFA, *v/v*, 15 min): t*_R_* = 12.7 min; K’ = 5.0.
**RP-HPLC (preparative):** (33-50% MeCN/H_2_O with 0.1% TFA, *v/v*, 20 min): t*_R_* = 18.5 min; K’ = 5.5.
**MS** **(ESI positive):** *m/z* calculated for C_90_H_133_FN_17_O_21_S_2_Si^+^: 1898.91, found: 634.0 [M+3H]^3+^, 950.3 [M+2H]^2+^, 1899.8 [M+H]^+^.

**[^nat^Ga]rhTATE2:***N*-SiFA*lin*-*N,N*-Me_2_-Gly-d-Lys(*trans*-[^nat^Ga]DOTA-TATE)-OH
**RP-HPLC (analytical):** (10-60% MeCN/H_2_O with 0.1% TFA, *v/v*, 15 min): t*_R_* = 13.2 min; K’ = 5.3.
**MS** **(ESI positive):** *m/z* calculated for C_90_H_131_FGaN_17_O_21_S_2_Si^+^: 1965.82, found: 656.2 [M+3H]^3+^, 983.8 [M+2H]^2+^, 1311.8 [2M+3H]^3+^.

**rhTATE2.1:**H-d-Cit-d-Cit-d-Cit-d-Dap(*N*-SiFA*lin*-*N,N*-Me_2_-Gly)-d-Lys(*trans*-DOTA-TATE)-OH
**RP-HPLC (analytical):** (10-60% MeCN/H_2_O with 0.1% TFA, *v/v*, 15 min): t*_R_* = 11.2 min; K’ = 4.3.
**RP-HPLC (preparative):** (33-50% MeCN/H_2_O with 0.1% TFA, *v/v*, 20 min): t*_R_* = 15.9 min; K’ = 5.1.
**MS** **(ESI positive):** *m/z* calculated for C_111_H_172_FN_28_O_28_S_2_Si^+^: 2456.21, found: 819.9 [M+3H]^3+^, 1229.1 [M+2H]^2+^, 1639.2 [2M+3H]^3+^, 1843.9 [3M+4H]^4+^.

**[^nat^Ga]rhTATE2.1:**H-d-Cit-d-Cit-d-Cit-d-Dap(*N*-SiFA*lin*-*N,N*-Me_2_-Gly)-d-Lys(*trans*-[^nat^Ga]DOTA-TATE)-OH
**RP-HPLC (analytical):** (10-60% MeCN/H_2_O with 0.1% TFA, *v/v*, 15 min): t*_R_* = 11.5 min; K’ = 4.5.
**MS** **(ESI positive):** *m/z* calculated for C_111_H_170_FGaN_28_O_28_S_2_Si^+^: 2523.12, found: 841.7 [M+3H]^3+^, 1262.3 [M+2H]^2+^, 1682.8 [2M+3H]^3+^.

**rhTATE2.2:**H-d-Glu-d-Cit-d-Cit-d-Dap(*N*-SiFA*lin*-*N,N*-Me_2_-Gly)-d-Lys(*trans*-DOTA-TATE)-OH
**RP-HPLC (analytical):** (10-60% MeCN/H_2_O with 0.1% TFA, *v/v*, 15 min): t*_R_* = 11.3 min; K’ = 4.4.
**RP-HPLC (preparative):** (33-40% MeCN/H_2_O with 0.1% TFA, *v/v*, 20 min): t*_R_* = 17.1 min; K’ = 5.4.
**MS** **(ESI positive):** *m/z* calculated for C_110_H_168_FN_26_O_29_S_2_Si^+^: 2428.17, found: 810.3 [M+3H]^3+^, 1215.2 [M+2H]^2+^, 1619.9 [2M+3H]^3+^.

**[^nat^Ga]rhTATE2.2:**H-d-Glu-d-Cit-d-Cit-d-Dap(*N*-SiFA*lin*-*N,N*-Me_2_-Gly)-d-Lys(*trans*-[^nat^Ga]DOTA-TATE)-OH
**RP-HPLC (analytical):** (10-60% MeCN/H_2_O with 0.1% TFA, *v/v*, 15 min): t*_R_* = 11.5 min; K’ = 4.5.
**MS** **(ESI positive):** *m/z* calculated for C_110_H_166_FGaN_26_O_29_S_2_Si^+^: 2495.08, found: 832.3 [M+3H]^3+^, 1248.3 [M+2H]^2+^, 1664.2 [2M+3H]^3+^.

**rhTATE2.3:**H-d-Glu-d-Glu-d-Dap-d-Dap(*N*-SiFA*lin*-*N,N*-Me_2_-Gly)-d-Lys(*trans*-DOTA-TATE)-OH
**RP-HPLC (analytical):** (10-60% MeCN/H_2_O with 0.1% TFA, *v/v*, 15 min): t*_R_* = 11.2 min; K’ = 4.3.
**RP-HPLC (preparative):** (30-43% MeCN/H_2_O with 0.1% TFA, *v/v*, 20 min): t*_R_* = 17.6 min; K’ = 5.8.
**MS** **(ESI positive):** *m/z* calculated for C_106_H_159_FN_23_O_29_S_2_Si^+^: 2329.09, found: 583.2 [M+4H]^4+^, 777.1 [M+3H]^3+^, 1165.2 [M+2H]^2+^, 1553.6 [2M+3H]^3+^, 1748.9 [3M+4H]^4+^.

**[^nat^Ga]rhTATE2.3:**H-d-Glu-d-Glu-d-Dap-d-Dap(*N*-SiFA*lin*-*N,N*-Me_2_-Gly)-d-Lys(*trans*-[^nat^Ga]DOTA-TATE)-OH
**RP-HPLC (analytical):** (10-60% MeCN/H_2_O with 0.1% TFA, *v/v*, 15 min): t*_R_* = 11.3 min; K’ = 4.4.
**MS** **(ESI positive):** *m/z* calculated for C_106_H_157_FGaN_23_O_29_S_2_Si^+^: 2396.00, found: 600.3 [M+4H]^4+^, 799.7 [M+3H]^3+^, 1199.1 [M+2H]^2+^, 1598.5 [2M+3H]^3+^.

**rhTATE2.4:**H-d-Glu-d-Glu-d-Lys-d-Dap(*N*-SiFA*lin*-*N,N*-Me_2_-Gly)-d-Lys(*trans*-DOTA-TATE)-OH
**RP-HPLC (analytical):** (10-60% MeCN/H_2_O with 0.1% TFA, *v/v*, 15 min): t*_R_* = 11.1 min; K’ = 4.3.
**RP-HPLC (preparative):** (30-47% MeCN/H_2_O with 0.1% TFA, *v/v*, 20 min): t*_R_* = 15.7 min; K’ = 4.7.
**MS** **(ESI positive):** *m/z* calculated for C_109_H_165_FN_23_O_29_S_2_Si^+^: 2371.13, found: 791.2 [M+3H]^3+^, 1186.8 [M+2H]^2+^, 1582.5 [2M+3H]^3+^.

**[^nat^Ga]rhTATE2.4:**H-d-Glu-d-Glu-d-Lys-d-Dap(*N*-SiFA*lin*-*N,N*-Me_2_-Gly)-d-Lys(*trans*-[^nat^Ga]DOTA-TATE)-OH
**RP-HPLC (analytical):** (10-60% MeCN/H_2_O with 0.1% TFA, *v/v*, 15 min): t*_R_* = 11.2 min; K’ = 4.3.
**MS** **(ESI positive):** *m/z* calculated for C_109_H_163_FGaN_23_O_29_S_2_Si^+^: 2438.04, found: 610.6 [M+4H]^4+^, 813.9 [M+3H]^3+^, 1220.3 [M+2H]^2+^, 1626.7 [2M+3H]^3+^.

**rhTATE2.5:**H-d-Glu-d-Glu-d-Glu-d-Dap(*N*-SiFA*lin*-*N,N*-Me_2_-Gly)-d-Lys(*trans*-DOTA-TATE)-OH
**RP-HPLC (analytical):** (10-60% MeCN/H_2_O with 0.1% TFA, *v/v*, 15 min): t*_R_* = 11.5 min; K’ = 4.5.
**RP-HPLC (preparative):** (30-43% MeCN/H_2_O with 0.1% TFA, *v/v*, 20 min): t*_R_* = 17.2 min; K’ = 5.3.
**MS** **(ESI positive):** *m/z* calculated for C_108_H_160_FN_22_O_31_S_2_Si^+^: 2372.08, found: 593.2 [M+4H]^4+^, 791.0 [M+3H]^3+^, 1185.9 [M+2H]^2+^, 1581.5 [2M+3H]^3+^, 1779.2 [3M+4H]^4+^.

**[^nat^Ga]rhTATE2.5:**H-d-Glu-d-Glu-d-Glu-d-Dap(*N*-SiFA*lin*-*N,N*-Me_2_-Gly)-d-Lys(*trans*-[^nat^Ga]DOTA-TATE)-OH
**RP-HPLC (analytical):** (10-60% MeCN/H_2_O with 0.1% TFA, *v/v*, 15 min): t*_R_* = 11.7 min; K’ = 4.6.
**MS** **(ESI positive):** *m/z* calculated for C_108_H_158_FGaN_22_O_31_S_2_Si^+^: 2438.99, found: 610.2 [M+4H]^4+^, 813.2 [M+3H]^3+^, 1219.2 [M+2H]^2+^, 1625.4 [2M+3H]^3+^, 1828.7 [3M+4H]^4+^.

**Supplementary table 1**: In vitro data for the 2^nd^ generation uncomplexed ligands.

| Name | Hydrophilic modifier | IC_50_ [nm] | LogD*_pH=7.4_* | HSA HPAC % |
| --- | --- | --- | --- | --- |
| **[^nat/18^F]rhTATE2.1** | d-Cit-d-Cit-d-Cit | 9.24±1.39 | ND | 93.6 |
| **[^nat/18^F]rhTATE2.2** | d-Cit-d-Cit-d-Glu | 10.6±1.01 | ND | 84.7 |
| **[^nat/18^F]rhTATE2.3** | d-Dap-d-Glu-d-Glu | 7.38±1.01 | −1.96 | 86.2 |
| **[^nat/18^F]rhTATE2.4** | d-Lys-d-Glu-d-Glu | 5.93±1.14 | −2.36 | 83.9 |
| **[^nat/18^F]rhTATE2.5** | d-Glu-d-Glu-d-Glu | 12.1±0.74 | ND | 84.8 |


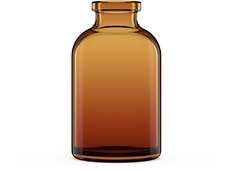


**VAC IN**

**6**

**7**

**8**

**9**

**10**

**MFC**

**γ2**

**VAC**

**γ3**

**γ1**

**3**

**4**

**5**

**2**

**1**

**1**

**3**

**4**

**5**

**2**

Final Vial

MeCN

Anhyd.

Elution

Cocktail

**250 mL Waste Bottle**


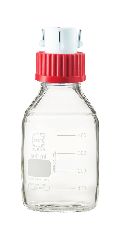


QMA

C18


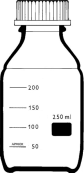


Reaction Vial

[^18^F]Fluoride

Target line

Buffer

EtOH:H_2_O

Eluent

**Supplementary Figure 4:** Layout of the cassette setup used for the ^18^F-fluorination of [^18^F][^nat^Ga]rhTATE2.5 on a Scintomics GRP^TM^ 3V module with two manifolds.


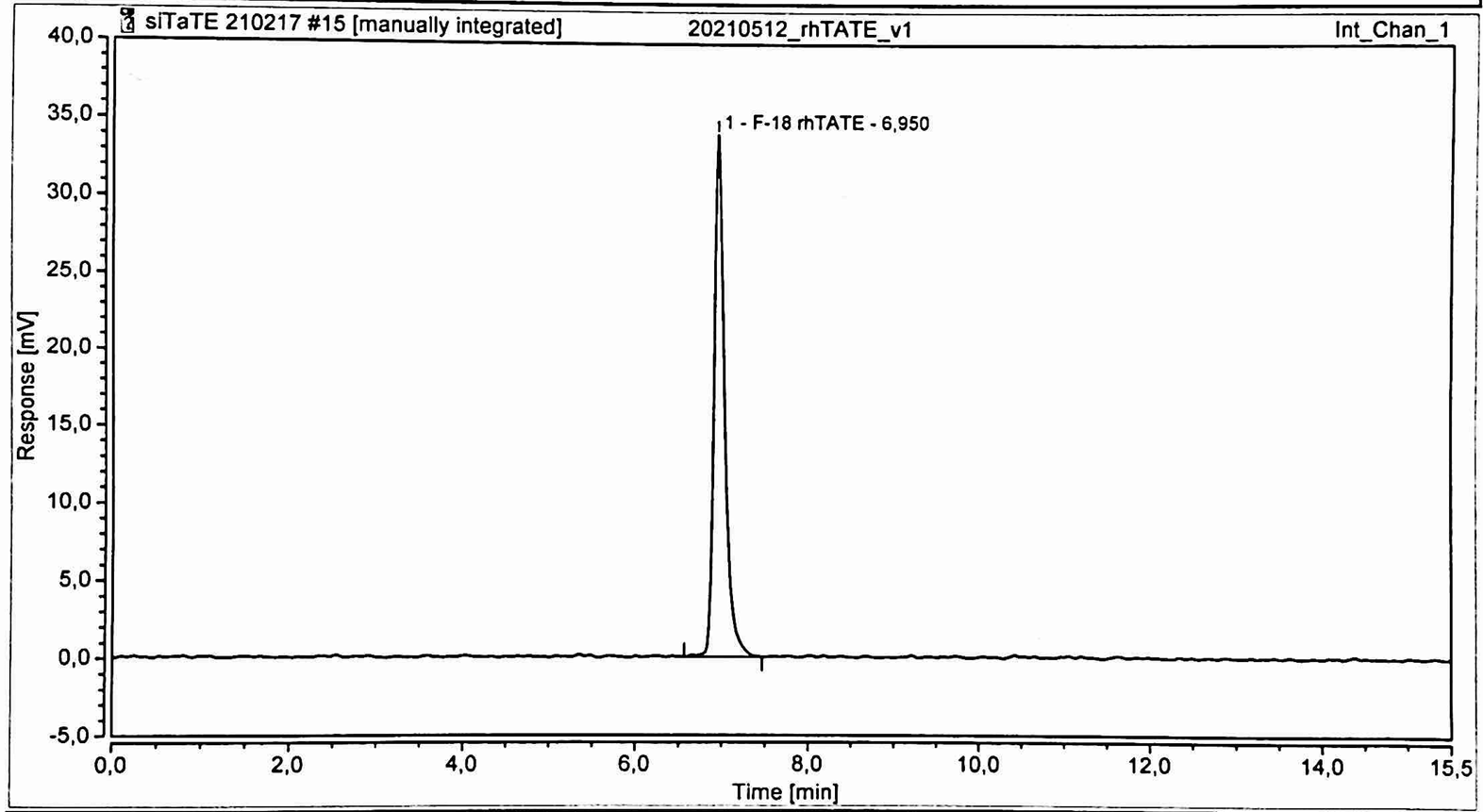


**1 – [^18^F][^nat^Ga]rhTATE2.5 – 6.950**

**Supplementary Figure 5:** RP-HPLC for the automatic ^18^F-fluorination of [^18^F][^nat^Ga]rhTATE2.5 (gradient of solvent B (MeCN with 0.1% TFA) in solvent A (H_2_O with 0.1% TFA; 0% B in A (0:00 min) → 5% B in A (0:30 min) → 80% B in A (10:30 min) → 5% B in A (15:30 min).


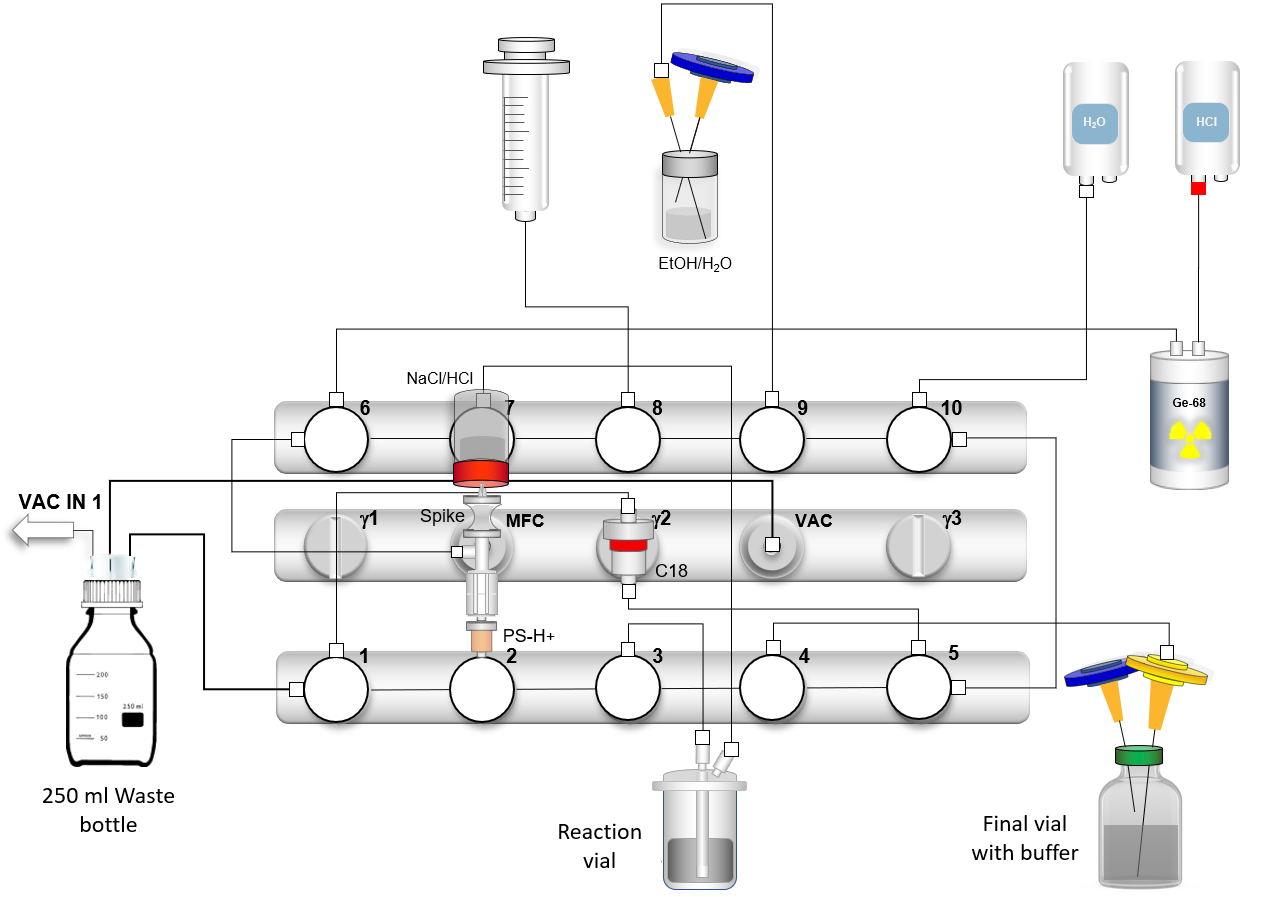


**Supplementary Figure 6:** Layout of the cassette setup used for the ^68^Ga-radiometallation of [^68^Ga]rhTATE2.5 on a Scintomics GRP^TM^ module with two manifolds.

**Supplementary table 2:** Results of the biodistribution studies (1h p.i.) of [^18^F][^nat^Ga]rhTATE2.2 and [^18^F][^nat^Ga]rhTATE2.5 compared to [^18^F]SiTATE. Values are given in %ID/g. p values are the results of unpaired Welch’s t-tests compared to [^18^F]SiTATE (p>0.05: n.d.; p≤0.05: *; p≤0.01: **;
p≤0.001: ***; p≤0.0001: ****).

|  | [^18^F]SiTATE  (n = 5) | [^18^F][^nat^Ga]rhTATE2.2  (n = 3) | | [^18^F][^nat^Ga]rhTATE2.5  (n = 4) | |
| --- | --- | --- | --- | --- | --- |
|  | Average ± Stdev | Average ± Stdev | p vs [^18^F]SiTATE | Average ± Stdev | p vs [^18^F]SiTATE |
| Blood | 0.81±0.11 | 0.39±0.19 | 0.044 | 0.32±0.11 | 0.0004 (***) |
| Heart | 0.47±0.06 | 0.33±0.01 | 0.006 (**) | 0.18±0.05 | 0.0001 (***) |
| Liver | 1.97±0.25 | 2.43±0.36 | 0.141 | 0.40±0.12 | 0.00002 (****) |
| Intestine | 1.67±0.21 | 2.87±0.15 | 0.00012 (***) | 1.30±0.25 | 0.056 |
| Muscle | 0.15±0.02 | 0.06±0.01 | 0.00016 (***) | 0.04±0.02 | 0.0001 (***) |
| Femur | 1.54±0.34 | 1.70±0.45 | 0.628 | 1.05±0.30 | 0.056 |
| Kidney | 16.30±1.00 | 32.60±3.50 | 0.012 | 12.50±3.50 | 0.116 |
| Adrenal  Glands | 1.89±0.37 | 4.35±0.69 | 0.014 | 1.52±0.21 | 0.104 |
| Lung | 3.38±0.62 | 6.29±0.65 | 0.003 (**) | 2.73±0.46 | 0.114 |
| Spleen | 0.66±0.08 | 1.31±0.20 | 0.022 | 0.35±0.07 | 0.0005 (***) |
| Stomach | 6.75±0.58 | 13.40±1.60 | 0.013 | 6.46±1.00 | 0.631 |
| Pancreas | 8.94±1.98 | 31.50±2.32 | 0.0002 (**) | 11.50±2.32 | 0.130 |
| Tumor | 22.70±2.68 | 27.90±4.78 | 0.190 | 18.60±6.18 | 0.285 |

**Supplementary table 3:** T/O ratios of the biodistribution studies (1h p.i.) of [^18^F][^nat^Ga]rhTATE2.2 and [^18^F][^nat^Ga]rhTATE2.5 compared to [^18^F]SiTATE. p values are the results of unpaired Welch’s t‑tests compared to [^18^F]SiTATE (p>0.05: n.d.; p≤0.05: *; p≤0.01: **; p≤0.001: ***; p≤0.0001: ****).

|  | [^18^F]SiTATE  (n = 5) | [^18^F][^nat^Ga]rhTATE2.2  (n = 3) | | [^18^F][^nat^Ga]rhTATE2.5  (n = 4) | |
| --- | --- | --- | --- | --- | --- |
|  | Average ± Stdev | Average ± Stdev | p vs [^18^F]SiTATE | Average ± Stdev | p vs [^18^F]SiTATE |
| Blood | 28.07±5.10 | 72.30±38.37 | 0.183 | 58.11±28.22 | 0.122 |
| Heart | 48.61±8.14 | 84.87±14.66 | 0.034 (*) | 100.97±44.51 | 0.098 |
| Liver | 11.53±2.02 | 11.47±2.60 | 0.975 | 46.79±20.85 | 0.042 (*) |
| Intestine | 13.58±2.34 | 9.73±1.74 | 0.041 (*) | 14.36±5.48 | 0.804 |
| Muscle | 150.65±23.73 | 460.20±86.68 | 0.021 (*) | 419.51±253.06 | 0.123 |
| Femur | 14.74±3.71 | 16.39±5.15 | 0.659 | 17.81±7.80 | 0.508 |
| Kidney | 1.40±0.19 | 0.86±0.17 | 0.0097 (**) | 1.49±0.64 | 0.801 |
| Adrenal  Glands | 12.00±2.74 | 6.41±1.49 | 0.0097 (**) | 12.24±4.41 | 0.928 |
| Lung | 6.72±1.47 | 4.43±0.89 | 0.034 (*) | 6.83±2.54 | 0.942 |
| Spleen | 34.21±5.95 | 21.29±4.89 | 0.020 (*) | 52.86±20.24 | 0.161 |
| Stomach | 3.37±0.49 | 2.08±0.43 | 0.012 (*) | 2.89±1.06 | 0.449 |
| Pancreas | 2.54±0.64 | 0.89±0.17 | 0.003 (**) | 1.62±0.63 | 0.070 |

**
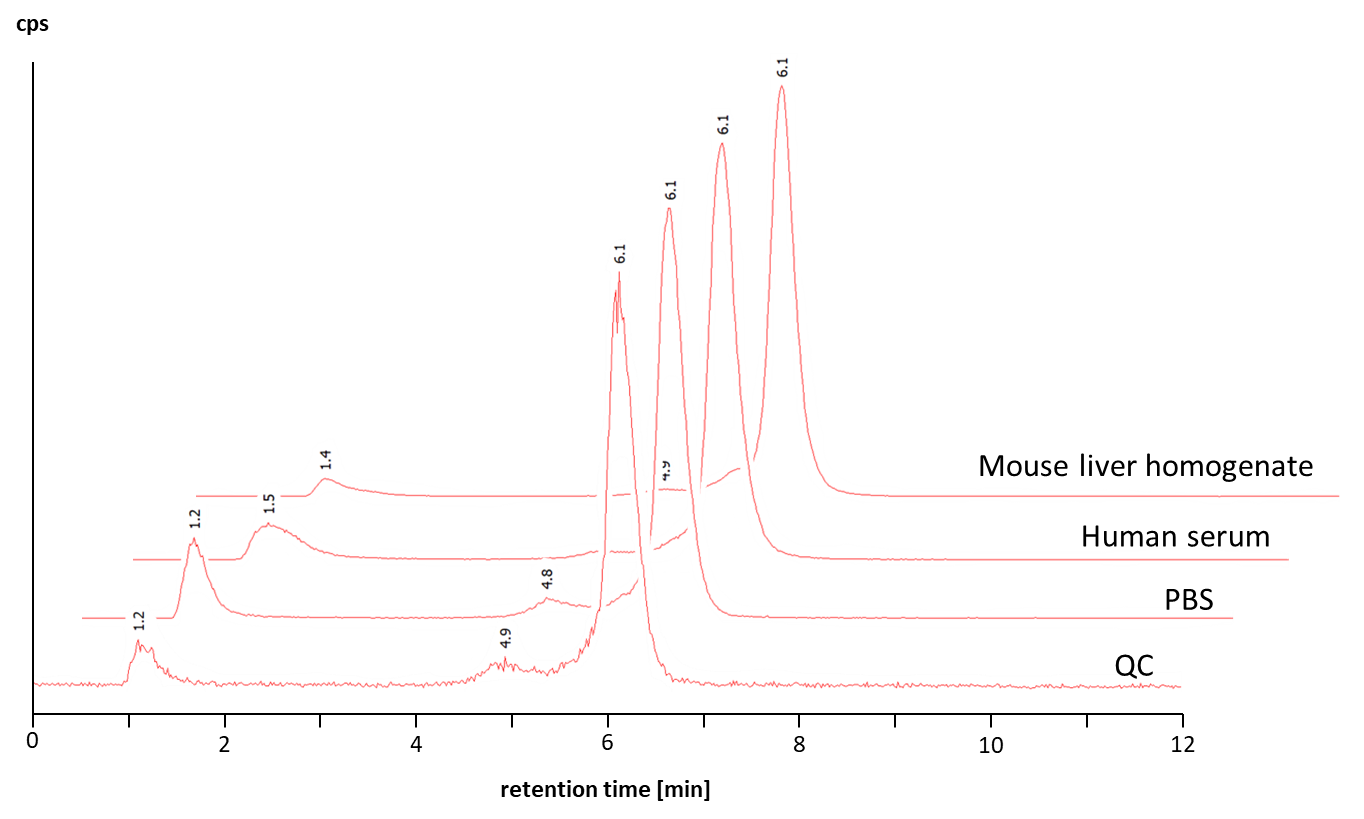
**

**Supplementary Figure 7:** Radio-HPLC traces of the quality control (QC) of freshly prepared [^nat^F][^68^Ga]rhTATE in comparison to [^nat^F][^68^Ga]rhTATE incubated with PBS, human serum, and mouse liver extract for 1h at 37°C.

**
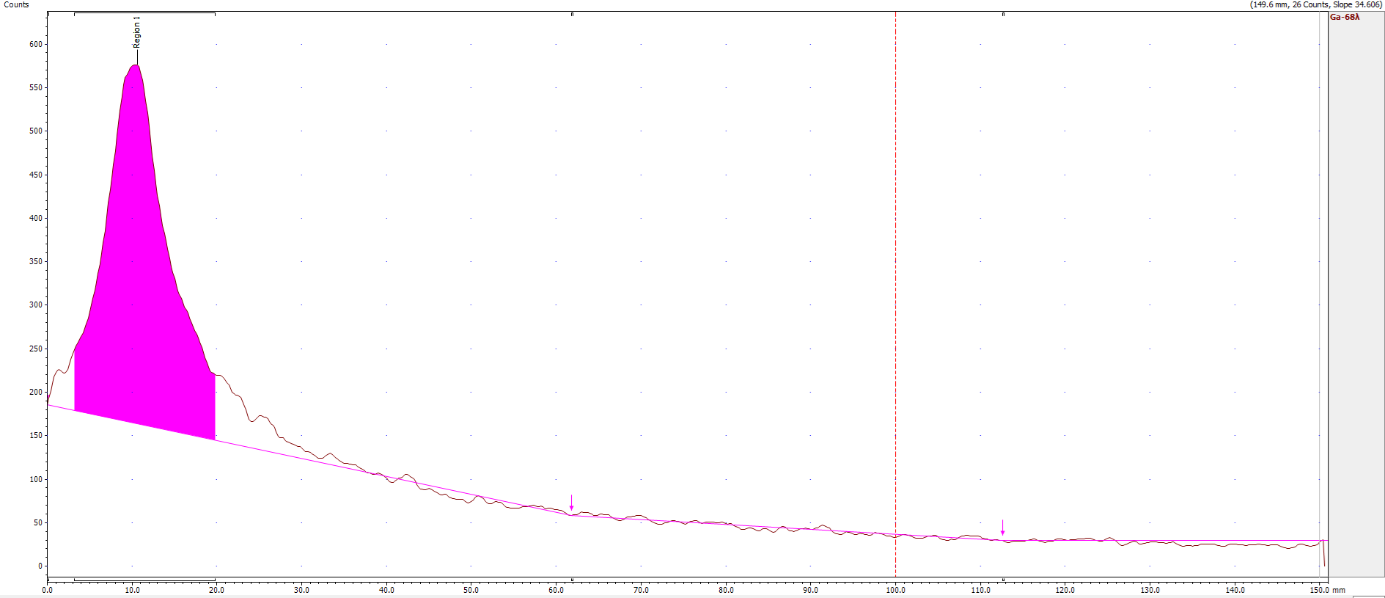
**

**Supplementary Figure 8:** Representative radio TLC of [^nat^F][^68^Ga]rhTATE, here after incubation with PBS (1h. 37°C). The radiolabeled peptide is retained at the start (R_f_=0.0-0.2) , while free ^68^Ga (R_f_=0.8-1.0) moves with the solvent front (no free ^68^Ga detected).
